# Supplementary material for: Behavioral insights during the COVID-19 pandemic in the Federation of Bosnia and Herzegovina: the role of trust, health literacy, risk and fairness perceptions in compliance with public health and social measures
Source: PLoS One. 2025 Apr 17;20(4):e0320433. doi: 10.1371/journal.pone.0320433 (PMC12005501; doi:10.1371/journal.pone.0320433)
Supplement: S1 Appendix — It also includes descriptive gender differences of self-reported pandemic behaviors. (DOCX) [file pone.0320433.s001.docx]

S1 Appendix: Regressions for Antecedents of pandemic behaviors during the 5 waves of data collection

| **Table S1. Risk Perceptions** | | | | | | | | | | | | | | | |
| --- | --- | --- | --- | --- | --- | --- | --- | --- | --- | --- | --- | --- | --- | --- | --- |
|  | **Wave 1** | | | **Wave 2** | | | **Wave 3** | | | **Wave 4** | | | **Wave 5** | | |
| *Predictors* | *Beta* | *standardized CI* | *p* | *Beta* | *standardized CI* | *p* | *Beta* | *standardized CI* | *p* | *Beta* | *standardized CI* | *p* | *Beta* | *standardized CI* | *p* |
| Age | 0.09 | 0.03 – 0.15 | **0.004** | 0.14 | 0.08 – 0.20 | **<0.001** | 0.18 | 0.12 – 0.24 | **<0.001** | 0.19 | 0.13 – 0.25 | **<0.001** | 0.17 | 0.11 – 0.24 | **<0.001** |
| male (vs. female) | -0.12 | -0.24 – -0.00 | **0.043** | -0.09 | -0.21 – 0.03 | 0.157 | -0.14 | -0.26 – -0.02 | **0.019** | -0.23 | -0.35 – -0.11 | **<0.001** | -0.13 | -0.25 – -0.00 | **0.045** |
| Education high (vs. low) | -0.09 | -0.55 – 0.38 | 0.709 | 0.13 | -0.33 – 0.59 | 0.577 | -0.67 | -1.18 – -0.17 | **0.009** | -0.83 | -1.30 – -0.36 | **0.001** | -0.06 | -0.57 – 0.44 | 0.809 |
| Working in Healthcare | 0.02 | -0.10 – 0.15 | 0.702 | 0.30 | 0.08 – 0.52 | **0.009** | 0.20 | 0.01 – 0.40 | **0.041** | 0.11 | -0.09 – 0.32 | 0.286 | -0.00 | -0.22 – 0.21 | 0.969 |
| Own children (in their household) | 0.09 | -0.03 – 0.21 | 0.141 | 0.18 | 0.06 – 0.30 | **0.004** | 0.04 | -0.08 – 0.16 | 0.512 | 0.10 | -0.03 – 0.22 | 0.122 | 0.14 | 0.01 – 0.27 | **0.035** |
| urban (vs. rural) | 0.16 | 0.04 – 0.28 | **0.009** | 0.13 | 0.01 – 0.26 | **0.032** | 0.13 | 0.01 – 0.26 | **0.040** | -0.01 | -0.13 – 0.11 | 0.864 | -0.00 | -0.13 – 0.12 | 0.969 |
| Observations | 1054 | | | 1000 | | | 1067 | | | 1068 | | | 1006 | | |
| R^2^ / R^2^ adjusted | 0.024 / 0.019 | | | 0.045 / 0.040 | | | 0.052 / 0.047 | | | 0.062 / 0.057 | | | 0.033 / 0.027 | | |

| **Table S2. Self-efficacy** | | | | | | | | | | | | |
| --- | --- | --- | --- | --- | --- | --- | --- | --- | --- | --- | --- | --- |
|  | **Wave 1** | | | **Wave 2** | | | **Wave 3** | | | **Wave 4** | | |
| *Predictors* | *Beta* | *standardized CI* | *p* | *Beta* | *standardized CI* | *p* | *Beta* | *standardized CI* | *p* | *Beta* | *standardized CI* | *p* |
| Age | 0.01 | -0.05 – 0.07 | 0.718 | 0.06 | -0.00 – 0.12 | 0.061 | -0.03 | -0.09 – 0.03 | 0.392 | -0.01 | -0.07 – 0.06 | 0.846 |
| male (vs. female) | 0.11 | -0.01 – 0.23 | 0.070 | 0.02 | -0.10 – 0.15 | 0.709 | 0.07 | -0.05 – 0.19 | 0.268 | -0.01 | -0.13 – 0.12 | 0.932 |
| Education high (vs. low) | 0.61 | 0.14 – 1.07 | **0.011** | 0.13 | -0.34 – 0.59 | 0.596 | 0.05 | -0.47 – 0.56 | 0.863 | 0.55 | 0.07 – 1.03 | **0.026** |
| Working in Healthcare | -0.06 | -0.18 – 0.06 | 0.346 | -0.35 | -0.58 – -0.13 | **0.002** | -0.04 | -0.24 – 0.16 | 0.704 | -0.10 | -0.31 – 0.11 | 0.365 |
| Own children (in their household) | -0.22 | -0.34 – -0.10 | **<0.001** | -0.02 | -0.14 – 0.11 | 0.788 | -0.10 | -0.22 – 0.02 | 0.117 | -0.12 | -0.24 – 0.01 | 0.071 |
| urban (vs. rural) | 0.02 | -0.10 – 0.14 | 0.754 | -0.12 | -0.24 – 0.01 | 0.066 | -0.07 | -0.20 – 0.06 | 0.268 | 0.05 | -0.07 – 0.18 | 0.427 |
| Observations | 1054 | | | 1000 | | | 1067 | | | 1068 | | |
| R^2^ / R^2^ adjusted | 0.022 / 0.016 | | | 0.017 / 0.011 | | | 0.006 / -0.000 | | | 0.009 / 0.003 | | |

| **Table S3. Trust in institutions** | | | | | | | | | | | | | | | |
| --- | --- | --- | --- | --- | --- | --- | --- | --- | --- | --- | --- | --- | --- | --- | --- |
|  | **Wave 1** | | | **Wave 2** | | | **Wave 3** | | | **Wave 4** | | | **Wave 5** | | |
| *Predictors* | *Beta* | *standardized CI* | *p* | *Beta* | *standardized CI* | *p* | *Beta* | *standardized CI* | *p* | *Beta* | *standardized CI* | *p* | *Beta* | *standardized CI* | *p* |
| Age | -0.00 | -0.06 – 0.06 | 0.953 | 0.03 | -0.04 – 0.09 | 0.414 | 0.00 | -0.06 – 0.06 | 0.983 | 0.03 | -0.03 – 0.09 | 0.369 | 0.01 | -0.06 – 0.07 | 0.848 |
| male (vs. female) | 0.06 | -0.06 – 0.18 | 0.310 | -0.13 | -0.25 – -0.00 | **0.047** | 0.11 | -0.01 – 0.23 | 0.069 | 0.01 | -0.11 – 0.13 | 0.877 | 0.13 | 0.00 – 0.25 | **0.044** |
| Education high (vs. low) | -0.04 | -0.51 – 0.43 | 0.862 | 0.29 | -0.18 – 0.77 | 0.229 | -0.12 | -0.66 – 0.41 | 0.646 | -0.15 | -0.65 – 0.34 | 0.550 | 0.43 | -0.08 – 0.94 | 0.100 |
| Working in Healthcare | 0.04 | -0.08 – 0.16 | 0.525 | 0.59 | 0.36 – 0.81 | **<0.001** | 0.31 | 0.10 – 0.51 | **0.003** | 0.34 | 0.13 – 0.55 | **0.002** | 0.37 | 0.15 – 0.58 | **0.001** |
| Own children (in their household) | 0.02 | -0.10 – 0.15 | 0.714 | 0.06 | -0.06 – 0.19 | 0.312 | -0.03 | -0.15 – 0.09 | 0.614 | -0.05 | -0.17 – 0.08 | 0.469 | -0.01 | -0.14 – 0.12 | 0.890 |
| urban (vs. rural) | -0.00 | -0.12 – 0.12 | 0.972 | -0.00 | -0.13 – 0.12 | 0.975 | -0.02 | -0.15 – 0.10 | 0.710 | -0.02 | -0.14 – 0.11 | 0.796 | 0.01 | -0.12 – 0.13 | 0.894 |
| Observations | 1054 | | | 989 | | | 1058 | | | 1057 | | | 999 | | |
| R^2^ / R^2^ adjusted | 0.002 / -0.004 | | | 0.034 / 0.028 | | | 0.012 / 0.006 | | | 0.011 / 0.005 | | | 0.017 / 0.011 | | |

| **Table S4. Emotional pandemic response** | | | | | | | | | | | | | | | |
| --- | --- | --- | --- | --- | --- | --- | --- | --- | --- | --- | --- | --- | --- | --- | --- |
|  | **Wave 1** | | | **Wave 2** | | | **Wave 3** | | | **Wave 4** | | | **Wave 5** | | |
| *Predictors* | *Beta* | *standardized CI* | *p* | *Beta* | *standardized CI* | *p* | *Beta* | *standardized CI* | *p* | *Beta* | *standardized CI* | *p* | *Beta* | *standardized CI* | *p* |
| Age | 0.08 | 0.01 – 0.14 | **0.015** | 0.05 | -0.01 – 0.11 | 0.110 | 0.10 | 0.04 – 0.16 | **0.002** | 0.16 | 0.10 – 0.22 | **<0.001** | 0.11 | 0.05 – 0.18 | **0.001** |
| male (vs. female) | -0.13 | -0.25 – -0.01 | **0.030** | -0.25 | -0.37 – -0.12 | **<0.001** | -0.04 | -0.16 – 0.08 | 0.476 | -0.10 | -0.22 – 0.02 | 0.095 | -0.06 | -0.19 – 0.06 | 0.327 |
| Education high (vs. low) | 0.17 | -0.30 – 0.64 | 0.474 | -0.21 | -0.67 – 0.26 | 0.382 | -0.10 | -0.61 – 0.41 | 0.700 | -1.04 | -1.51 – -0.57 | **<0.001** | 0.20 | -0.31 – 0.71 | 0.442 |
| Working in Healthcare | -0.02 | -0.14 – 0.10 | 0.718 | 0.03 | -0.19 – 0.26 | 0.779 | 0.12 | -0.08 – 0.32 | 0.235 | 0.26 | 0.06 – 0.47 | **0.012** | -0.06 | -0.27 – 0.16 | 0.615 |
| Own children (in their household) | 0.07 | -0.06 – 0.19 | 0.285 | 0.05 | -0.08 – 0.17 | 0.445 | 0.13 | 0.01 – 0.25 | **0.036** | 0.06 | -0.06 – 0.18 | 0.324 | 0.03 | -0.10 – 0.16 | 0.680 |
| urban (vs. rural) | 0.05 | -0.07 – 0.17 | 0.390 | 0.17 | 0.05 – 0.29 | **0.007** | 0.07 | -0.06 – 0.20 | 0.289 | -0.10 | -0.22 – 0.03 | 0.122 | 0.16 | 0.04 – 0.29 | **0.010** |
| Observations | 1054 | | | 1000 | | | 1067 | | | 1068 | | | 1006 | | |
| R^2^ / R^2^ adjusted | 0.014 / 0.008 | | | 0.028 / 0.023 | | | 0.016 / 0.010 | | | 0.047 / 0.042 | | | 0.023 / 0.017 | | |

| **Table S5. Health Literacy** | | | | | | | | | | | | |
| --- | --- | --- | --- | --- | --- | --- | --- | --- | --- | --- | --- | --- |
|  | **Wave 2** | | | **Wave 3** | | | **Wave 4** | | | **Wave 5** | | |
| *Predictors* | *Beta* | *standardized CI* | *p* | *Beta* | *standardized CI* | *p* | *Beta* | *standardized CI* | *p* | *Beta* | *standardized CI* | *p* |
| Age | 0.05 | -0.01 – 0.11 | 0.112 | -0.00 | -0.06 – 0.06 | 0.928 | 0.01 | -0.05 – 0.07 | 0.797 | 0.04 | -0.03 – 0.10 | 0.268 |
| male (vs. female) | -0.08 | -0.20 – 0.05 | 0.225 | 0.12 | -0.00 – 0.24 | 0.059 | -0.02 | -0.14 – 0.10 | 0.751 | 0.03 | -0.09 – 0.16 | 0.634 |
| Education high (vs. low) | 0.02 | -0.45 – 0.49 | 0.936 | -0.19 | -0.71 – 0.32 | 0.459 | 0.11 | -0.37 – 0.59 | 0.649 | -0.14 | -0.65 – 0.37 | 0.586 |
| Working in Healthcare | 0.11 | -0.12 – 0.34 | 0.338 | 0.02 | -0.18 – 0.22 | 0.839 | 0.23 | 0.02 – 0.45 | **0.030** | 0.19 | -0.03 – 0.41 | 0.088 |
| Own children (in their household) | 0.07 | -0.06 – 0.19 | 0.296 | 0.08 | -0.04 – 0.20 | 0.196 | -0.03 | -0.15 – 0.10 | 0.684 | -0.08 | -0.21 – 0.05 | 0.249 |
| urban (vs. rural) | -0.07 | -0.20 – 0.05 | 0.269 | -0.08 | -0.21 – 0.05 | 0.219 | -0.00 | -0.13 – 0.12 | 0.950 | 0.03 | -0.10 – 0.15 | 0.664 |
| Observations | 1000 | | | 1067 | | | 1068 | | | 1006 | | |
| R^2^ / R^2^ adjusted | 0.008 / 0.002 | | | 0.007 / 0.001 | | | 0.005 / -0.001 | | | 0.007 / 0.001 | | |

| **Table S6. Policy acceptance** | | | | | | | | | | | | | | | |
| --- | --- | --- | --- | --- | --- | --- | --- | --- | --- | --- | --- | --- | --- | --- | --- |
|  | **Wave 1** | | | **Wave 2** | | | **Wave 3** | | | **Wave 4** | | | **Wave 5** | | |
| *Predictors* | *Beta* | *standardized CI* | *p* | *Beta* | *standardized CI* | *p* | *Beta* | *standardized CI* | *p* | *Beta* | *standardized CI* | *p* | *Beta* | *standardized CI* | *p* |
| Age | 0.05 | -0.01 – 0.11 | 0.121 | 0.01 | -0.05 – 0.08 | 0.677 | 0.09 | 0.03 – 0.15 | **0.004** | 0.13 | 0.07 – 0.19 | **<0.001** | 0.12 | 0.06 – 0.19 | **<0.001** |
| male (vs. female) | -0.18 | -0.30 – -0.06 | **0.004** | -0.26 | -0.38 – -0.13 | **<0.001** | -0.05 | -0.17 – 0.07 | 0.402 | -0.28 | -0.40 – -0.16 | **<0.001** | -0.06 | -0.18 – 0.07 | 0.357 |
| Education high (vs. low) | 0.00 | -0.46 – 0.47 | 0.997 | 0.33 | -0.14 – 0.79 | 0.167 | 0.08 | -0.44 – 0.59 | 0.775 | 0.21 | -0.27 – 0.68 | 0.389 | 0.46 | -0.05 – 0.96 | 0.079 |
| Working in Healthcare | 0.00 | -0.12 – 0.12 | 0.987 | 0.12 | -0.10 – 0.35 | 0.276 | 0.05 | -0.15 – 0.25 | 0.652 | 0.09 | -0.12 – 0.29 | 0.416 | 0.15 | -0.07 – 0.36 | 0.182 |
| Own children (in their household) | 0.14 | 0.02 – 0.26 | **0.027** | 0.10 | -0.03 – 0.22 | 0.120 | 0.00 | -0.12 – 0.12 | 0.982 | 0.04 | -0.08 – 0.17 | 0.490 | 0.06 | -0.07 – 0.19 | 0.374 |
| urban (vs. rural) | 0.08 | -0.04 – 0.20 | 0.196 | 0.03 | -0.10 – 0.15 | 0.689 | 0.04 | -0.09 – 0.17 | 0.516 | -0.06 | -0.18 – 0.07 | 0.374 | -0.05 | -0.18 – 0.07 | 0.393 |
| Observations | 1054 | | | 1000 | | | 1067 | | | 1068 | | | 1006 | | |
| R^2^ / R^2^ adjusted | 0.018 / 0.013 | | | 0.023 / 0.017 | | | 0.009 / 0.004 | | | 0.039 / 0.034 | | | 0.019 / 0.013 | | |

**Table S7. Descriptive gender differences of self-reported protective behaviors.**

| Preventive behavior | Female |  |  | Male |  |  | Total |  |  |
| --- | --- | --- | --- | --- | --- | --- | --- | --- | --- |
| *(n, %)* | Yes | No | Does not apply | Yes | No | Does not apply | Yes | No | Does not apply |
| Wearing a face mask | 2041 (48.7%*) | 112 (2.7%*) | 15 (0.4%*) | 1805 (43.1%*) | 174 (4.2%*) | 42 (1.0%) | 3846 (91.8%) | 286 (6.8%) | 57 (1.4%) |
| Physical distance (2 m) | 1970 (47.0%*) | 140 (3.3%*) | 58 (1.4%*) | 1747 (41.7%*) | 208 (5.0%*) | 66 (1.6%) | 3717 (88.7%) | 348 (8.3%) | 124 (3.0%) |
| Avoiding touching eyes, nose, and mouth with unwashed hands | 1974 (47.1%*) | 139 (3.3%*) | 55 (1.3%*) | 1693 (40.4%*) | 236 (5.6%*) | 92 (2.2%) | 3667 (87.5%) | 375 (9.0%) | 147 (3.5%) |
| Disinfecting surfaces | 1808 (43.2%*) | 301 (7.2%*) | 59 (1.4%*) | 1504 (35.9%*) | 440 (10.5%*) | 77 (1.8%) | 3312 (79.1%) | 741 (17.7%) | 136 (3.2%) |
| Use of antibiotics when being sick | 243 (5.8%*) | 1723 (41.1%*) | 202 (4.8%*) | 290 (6.9%*) | 1581 (37.7%*) | 150 (3.6%) | 533 (12.7%) | 3304 (78.9%) | 352 (8.4%) |

*Percentage of the total
