# Supplementary material for: Behavioral insights during the COVID-19 pandemic in the Federation of Bosnia and Herzegovina: the role of trust, health literacy, risk and fairness perceptions in compliance with public health and social measures
Source: PLoS One. 2025 Apr 17;20(4):e0320433. doi: 10.1371/journal.pone.0320433 (PMC12005501; doi:10.1371/journal.pone.0320433)
Supplement: Attachment — Submitted filename: 2024_PHSM_Response_R1.docx [file pone.0320433.s003.docx]

Dear Editors, dear Reviewers,

We would like to express our sincere gratitude for your invaluable feedback and suggestions on our manuscript. Your thorough review and insightful comments have significantly contributed to improving the clarity and overall quality of our work.

We are pleased to inform you that we have addressed all of your recommendations in the revised manuscript. The detailed responses to each comment are displayed in a point-by-point response below.

We believe the revisions we made have significantly enhanced the manuscript. The improvements have clarified the key points, refined the methodology, and strengthened the overall argument. We hope that the revised manuscript is now suitable for publication and look forward to your positive response.

Reviewer #1:

Abstract:

**The transition between sections could be smoother.**

>>We included some changes in the abstract, including the shift and integration of the last sentence from the background section to the materials and methods section, connecting the two sections by re-acknowledging the studies goals:

“This study examines self-reported compliance with PHSM during the COVID-19 pandemic in the Federation of Bosnia and Herzegovina (FBIH).
**Materials and methods**: We analyze the association between compliance and behavioral determinants, using data from five cross-sectional surveys that were conducted between June 2020 and August 2021 in FBIH. Data from 1000 people per wave were collected, with participants being representative of FBIH regarding age, sex, and education level based on the data from the latest census in Bosnia and Herzegovina. (p. 2, l.39-46)

**- Consider reordering phrases like: "The present study provides behavioral insights about self-reported compliance with PHSM" could be simplified to "This study examines self-reported compliance with PHSM," reducing wordiness.**

>> We changed the respective sentence in accordance with the reviewer’s suggestion:

“This study examines self-reported compliance with PHSM during the COVID-19 pandemic in the Federation of Bosnia and Herzegovina (FBIH).” (p. 2, l. 39-41)

**- The description of the sample size ("1000 people representative of FBIH...") could mention how the representativeness was ensured. Did you use specific quotas for age, sex, and education level?**

>> We specified which census data was used for quota-based sampling:

“We analyze the association between compliance and behavioral determinants, using data from five cross-sectional surveys that were conducted between June 2020 and August 2021 in FBIH. Quota-based sampling ensured that the 1000 people per wave were population representative regarding age, sex, and education level based on the data from the latest census in Bosnia and Herzegovina. “ (p. 2, l. 42-41)

**- You might also want to clarify what "cross-sectional studies" means in this context. Was data collection repeated in different periods (longitudinal cross-sectional design), or was it one cross-sectional study with waves?**

>> We included the information that 1000 people *per wave* were included and hope, to the limited word-count in the abstract, that this clarifies the data collection method.
For the respective sentence, please find the changes in the reference above.

**- Avoid ambiguous terms like "key drivers" unless they are defined explicitly.**

**-It would help to specify which variables were strongest in the regression model and provide some context for the strength of associations (e.g., R-squared values)**

>> We changed key drivers to significant predictors in the abstract. We also included the minimum and maximum standardized beta-weights from the five regression analyses behind every predictor to indicate minimum and maximum explanatory power of the predictors:

“In five wave-specific regression analyses, emotional response (*β_min/max_* = .11*/.21*), risk perception (*β_min/max_* = .06/.18*), policy acceptance (*β_min/max_* = .09/.20*), and trust in institutions (*β_min/max_* = .06/.21*) emerged as significant predictors of PHSM.“ (p. 2, l.53-55)

**- The suggestion to support health literacy in specific groups could benefit from specifying what groups were most vulnerable or poorly informed based on the findings.**

>> Due to the world limit for abstracts we needed to shorten the abstract, and we decided for a general recommendation for health literacy support here:

“The study also affirms the impact of public trust on compliance, the risk of eroding compliance over time, and the need for health literacy support to help reinforce protective behaviors. (p. 2, l.59)

Introduction:

**- The opening sentence could be clearer and more engaging. Instead of starting with “Behavioral insights during the COVID-19 pandemic 60 in the Federation of Bosnia and Herzegovina,” consider starting with a broader statement about the importance of understanding population behaviors during health crises.**

>> As we formatted our manuscript following the APA 7 guidelines, we included the Title of the manuscript again as title for the introduction. However, as we re-read the guidelines for PLOS-One, we see that other headlines are allowed as well. We thank the reviewer #1 for this recommendation, and chose the title “Knowing How, Knowing Why: A behavioral perspective on public health and social measures” for our introduction. (p. 3, l.61)

**-Clarity in Purpose:**

**The introduction could benefit from explicitly stating early on why understanding these behaviors matters for the success of PHSM, tying this more closely to the study being introduced.**

**Suggested revision: This study aimed to assess the behavioral determinants that influence compliance with PHSM in the Federation of Bosnia and Herzegovina (FBIH) during the COVID-19 pandemic.**

>> We included the general aim of this study at the end of the first paragraph of the introduction. We added the time frame of data collection, because we did not analyze data from following years.

“Risk perception and behaviors are essential for understanding compliance with PHSM, but in turn, they are influenced by several other factors, including individuals’ perceptions of the consistency, competence, fairness, and objectivity of governmental authorities (4,5,6). This study aimed to assess the behavioral determinants that influence compliance with PHSM in the Federation of Bosnia and Herzegovina (FBIH) during the first year of the COVID-19 pandemic 2020-2021. (p. 3, l.68-70)

**- The transition from the general discussion of PHSM to Bosnia’s specific context could be made smoother.**

>> We now included the beforementioned concepts of PHSM policies and behavioral research before introducing the studies context in the introduction:

“Both PHSM policies, such as cancellation of mass gatherings and complementary behavioral research monitorings were implemented in the Federation of Bosnia and Herzegovina (FBIH), tailored and adapted to the specific epidemiological situation that changed over the course of the pandemic (9,10,11). (p 3, l.78-80)

**- The mention of the WHO PHSM recommendations and the survey tool implementation in Bosnia appears twice. It could be streamlined to avoid repetition.**

>> We shortened the last paragraph of the introduction, including the information about PHSM and BI studies in the paragraph above:

„ The aims of the survey were to 1) monitor the behavioral determinants critical for population compliance with the PHSM, including health literacy, risk perceptions, trust, and perception of fairness of the measures; 2) assess population behavioral changes regarding uptake of PHSM over time; 3) see how those changes relate to behavioral determinants, and contribute to findings on factors encouraging PHSM uptake more broadly; and 4) identify socio-demographic factors (such as sex and age) that help to describe target groups with low compliance and low trust.

This study scrutinizes the results of the behavioral monitoring system in conjunction with self-reported behaviors. The paper begins by outlining the research methodology including the survey measures. Section II analyses the survey data across the five waves. Section III discusses the key behavioral determinants of PHSM uptake in FBIH. Section IV concludes and offers recommendations.“ (p. 4, l. 84-93)

Methods:

**The sentence starting with "Time of data collection" (lines 96-98) is a bit awkward and can be restructured for better readability. It might flow better if the dates for each wave are listed in a tabular format or set apart as bullet points.**

>> We included the reviewers suggestion and changed the sentence into a list of bullet points, clearly improving the paragraphs readability:

“ **Sample and procedure**

The research team fielded five cross-sectional survey waves in the FBIH during different periods of the COVID-19 pandemic in the country between June 2020 and August 2021, reaching a total of N=5,195 participants, approximately 1,000 unique respondents per wave (12).

**Time of data collection:**

-first wave 05/06/2020 - 08/06/2020;

-second wave 03/07/2020 - 06/07/2020;

-third wave 19/09/2020 - 22/09/2020;

-fourth wave 04/12/2020 - 07/12/2020; and

-fifth wave 05/08/2021 - 08/08/2021. “ (p.4, l. 99-104)

**-The response rate section (lines 102-105) is informative but could be made clearer with some rewording. The phrasing feels slightly redundant and could use more precision in presenting the varying rates.**

>> We rephrased the sentences, and now state more precisely:

„The research company collecting the data distributed the online questionnaires to various population segments, enabling collection of a diverse range of respondents over time. While distribution methods were constant, response rates increased in the later waves (Wave 1: 21%, 4.818 invitations; Wave 4: 54%, 1.958 invitations; Wave 5: 47%, 2.158 invitations).” (p. 5, l. 117-119)

**- The sentence "Dropout rate for all five waves were 8%" (line 109) should have more context on what this means for the study. Does this reflect a good retention rate? Additionally, "were" should be corrected to "was."**

>> We clarified that we included participants in the dropout count, that ended the questionnaire before giving all answers now in the manuscript:

“Participants were allowed to end the questionnaire at any time. However, over all five waves only 8% of participants who started the questionnaires dropped out before giving all answers.” (p.5, l.123-124)

**- It would help to clarify why there was such a variation in the number of people invited to each wave and the response rates. It might indicate something about the survey methodology or external factors.**

>> As we reported these invitation numbers, we decided to discuss possible consequences for the data interpretation (and further recommendations for future studies) in the limitations section of the data:

As a further limitation, we experienced a difference in the invitation / participation ratio over the waves, with more people needed to be invited at the earlier waves of the cross-sectional survey. This might be caused by the intense information density at the earlier stages of the pandemic. In consequence, people may have been striving more for distraction, producing a self-selection bias in the early waves of our data. The people who were affected most strongly by the pandemic may have evaded the questionnaire study. Future pandemic studies with special panels could carefully address precisely these people, e.g. with help hotlines or counseling services, including qualitative approaches alongside the quantitative data for the general population. With this approach, behavioral insights could also be used for this target group to adapt pandemic decisions and support even better to the people who are psychologically affected most. (p.27, l. 534-543)

**- Remove unnecessary details: For example, in the "Health literacy" section, you mention internal consistency in both waves, but this could be summarized without repeating the Cronbach's alpha scores twice.**

>> We shortened the respective paragraph to:

“Health literacy. In waves 2-4, participants were asked about their perceived ability to find the information they needed about symptoms, treatments, and behavioral recommendations for COVID-19. In wave 5, this scale was extended with six items to COVID-19 vaccination literacy. Respondents rated their agreement on a 7-point scale ranging from 1 (strongly disagree) to 7 (strongly agree). Cronbach’s alpha indicates excellent internal consistency (α =.88 [.88;.89]) in all waves. (p.6-7; l. 154-162)

>>Also, we deleted the sentences about predictors and determinants from every paragraph in the methods section, because we include the description paragraph about the statistical analyses, avoiding repetition.

**- Avoid over-explaining Likert scales: In multiple instances, the explanation of Likert scales (1 to 7) could be simplified. After the first explanation, the reader should understand how the scale works, so reiterating it for every variable is unnecessary.**

>> We agree with the reviewer that explaining the scales is bringing a lot of repetition In the methods section of the manuscript. We can simplify the sentences by deleting the “7-point” information. However, we would strongly argument for including the description of the endpoints of the scales in the text for two reasons

(1) They change over the different predictors, having for example 1 spreading slow vs. 7 spreading fast; and 1 (strongly disagree) to 7 (strongly agree)
(2) They indicate what higher (or lower) mean values for participants indicate in terms of content, and they help interpret the direction of the effects (or example higher values mean higher trust and higher emotional response), being precise and replicable.

Given these two implications, we hope that the reviewer can agree with us in this compromise.

**- Separate statistical analysis section more clearly: While you do have a statistical analysis section (line 161), the explanations of statistical tests used, like ANOVA and multiple regression, are a bit disjointed. It might help to structure this as a formal, separate subsection that outlines the methodology in a clearer step-by-step manner.**

>> We now included a more separated structure for the different analyses. We start in each paragraph with the goal that this analysis procedure has and hope, this is now more understandable. The complete reworked paragraph can be seen under the following comment.

**- Explain the statistical tests more clearly: While you describe using one-way ANOVA and Tukey post-hoc tests, it could be helpful to include brief reasoning on why these tests were chosen (i.e., for comparing means across multiple groups) and how they were applied in the context of the study.**

>> we included brief descriptions for the statistical tests we used and also made the respective changes from the comment above:

**Statistical analysis**

First, descriptive statistics were calculated for all demographic characteristics and outcome variables to provide an overview of the sample.

Second, we aimed to examine whether the average values of behavioral determinants changed between the five survey waves. To do this, we performed six one-way analyses of variance (ANOVA), one for each behavioral determinant (self-efficacy, risk perceptions, emotional response, trust, health literacy, and fairness and policy acceptance). ANOVA is a statistical method used to compare means across multiple groups—in this case, the five survey waves—and determine if differences are statistically significant. Tukey post hoc tests were applied to identify specific pairwise differences between the cross-sectional samples. Additionally, we performed an ANOVA for protective behaviors to analyze whether they varied between the five waves.

Third, multiple linear regressions were used to identify the relationship between one dependent variable (e.g., preventive behaviors) and multiple independent variables (e.g., behavioral determinants). To further explore whether socio-demographic characteristics (predictors like age, gender and education) influenced behavioral determinants (dependent variables like trust or self-efficacy), additional linear regressions were conducted, with results detailed in Appendix 1. All analyses were performed using R-Studio, and the raw datasets are available for replication in an Open Science Repository. (p. 7, l.177-194)

**- Explain variable selection more clearly: You mention why certain items were excluded from certain scales (e.g., in the Emotional Response section). This could be more explicit, explaining the reasoning behind why a low correlation or Cronbach's alpha led to their removal. Additionally, why you chose to only focus on these specific seven variables should be justified more thoroughly in the introduction to this section.**

>> We included now more information about the scale origins and the rationale to exclude the items in the emotional response scale:

**Emotional response.** Emotions, especially fear, can play a significant role in motivating individuals to adopt prevention behaviors. According to Bradley & Lang (22), dominance, valence and arousal are important dimensions of emotional responses. We collected the emotional response to the pandemic situation with eight items. All were collected on semantic differentials (e.g., Perceiving the virus as 1 spreading slow vs. 7 spreading fast; Perceiving the situation as 1 not stressful vs. 7 extremely stressful). Two items, perceiving the pandemic as media-hyped (vs. not) and feeling depressed (vs. not) might cover the results of more complex cognitive and affective processes (23) rather than the initial emotional response dimensions. Reliability analyses showed that those items were not sufficiently correlated with the rest of the emotional response scale. To reach a sufficient Cronbach’s alpha (α =.71 [.70;.72]), the two items had to be excluded from the score. (p.6, l.148-157)

>> Also, we included explanations for the other behavioral determinants, such as self-efficacy:

**Self-efficacy.** Self-efficacy refers to an individual’s belief in their capacity to execute behaviors necessary to achieve specific outcomes (14). It is a critical factor in several theories of health behavior (15, 16) and has been shown to influence infection prevention (17,18). Two items were used to assess self-efficacy. The first item covered the theoretical efficacy (i.e., knowing how to protect oneself) ranging from 1 (not at all) to 7 (very much so). The second item covers the behavioral self-efficacy (i.e., avoiding an infection in the current situation) ranging from 1 (extremely difficult) to 7 (extremely easy). Due to a low correlation of the items (r=.32), we only use the behavioral self-efficacy as a behavioral determinant in the regression analyses. (p.5, l. 134-141)

Discussion:

**-The paragraph discussing health literacy (lines 412-423) could benefit from a more explicit connection between the data and its implications. You mention it was not a significant antecedent, but then delve into the details without clearly tying back to this point. A stronger conclusion for this section would enhance the overall coherence.**

>> We included now more information related stronger conclusion to health literacy (p 26, l 456-461):

“Health literacy was not a significant antecedent of predictive behaviors in our analysis. Although health literacy is repeatedly associated in the literature with people being better able to understand and apply more proactive health protection, there is no relationship between health literacy and PHSM in the multiple regression. The global pandemic and the associated media attention could have made it easier to find information, so that everyone had access to easily understandable information. In line with this explanation, the data for health literacy showed that information seeking was assessed as relatively straightforward, with individuals feeling capable of finding information about symptoms or regulations. In contrast, lower values were evident when it came to translating this information into daily life, indicating difficulties in incorporating regulations and recommendations into everyday decisions.”

**-Some sections, like the one on emotional response (lines 367-372), could use more precise connections between your data and the broader literature. You cite multiple studies, but a clearer explanation of how your results align or diverge from these findings could strengthen the analysis. For example, expand on how the spike in emotional response in the fourth wave specifically impacted protective behaviors in comparison to other waves.**

>> We included more information where our results are in line with international literature:

“Our findings on the predictive power of emotional responses align with broader literature from Europe, Asia and North America (29-34). In European countries, perceived threat and negative emotional reactions, such as worry, have been consistently identified as key predictors of self-reported preventive behaviors during the COVID-19 pandemic (31). Research shows that individuals perceiving greater threats and experiencing stronger negative emotions, including fear and anxiety, are more likely to adopt protective measures such as social distancing, mask-wearing, and adherence to health guidelines (30). Kim et al. found specifically that negative emotions were strong predictors of protective behaviors in Korea (33). Similarly, in a study by Liu et al., negative emotions increased risk perception, which in turn increased intentions to wear face masks in the US (34). (p. 24, l. 400-407)

Regarding COVID-19 risk perception, respondents initially exhibited a noticeable increase, which continued to rise steadily through the first four waves of data collection, culminating in a significant surge during the fourth wave. In the Federation of Bosnia and Herzegovina (FBiH), where the pandemic was marked by fluctuating case numbers, the population may have experienced fatigue in consistently adhering to preventive measures (28).The data indicate a pronounced increase in risk perception around weeks 44 to 49 in 2020 (corresponding with the fourth wave), which coincided with a high number of COVID-19 positive people and deaths (11). Notably, individuals living in urban areas or dealing with chronic illnesses tended to demonstrate heightened levels of risk perception, emphasizing the influence of environmental and health-related factors on perceived risk intensity. Our analysis revealed a positive correlation between risk perceptions and the increased adoption of protective behaviors, suggesting that heightened risk perception promotes the adoption of protective measures. (p 24-25, l. 408-418)

**- While you effectively reference several studies, you could integrate them more dynamically into your discussion. Rather than listing studies as standalone facts, try weaving their conclusions into your argument.**

>> we added findings from the respective studies:

>> Our findings on the predictive power of emotional responses align with broader literature from Europe, Asia and North America (29-34). In European countries, perceived threat and negative emotional reactions, such as worry, have been consistently identified as key predictors of self-reported preventive behaviors during the COVID-19 pandemic (31). Research shows that individuals perceiving greater threats and experiencing stronger negative emotions, including fear and anxiety, are more likely to adopt protective measures such as social distancing, mask-wearing, and adherence to health guidelines (30). Kim et al. found specifically that negative emotions were strong predictors of protective behaviors in Korea (33). Similarly, in a study by Liu et al., negative emotions increased risk perception, which in turn increased intentions to wear face masks in the US (34). (p. 24, l. 409-415)

**- The limitations section is well-acknowledged, but you could go deeper into how these limitations might have influenced your results. For instance, what specific elements of trust or health literacy might have been underrepresented due to sampling bias (lines 424-428)? Offering suggestions for future research or alternative methodologies could enhance the critical depth of this section.**

>> We included a more detailed discussion about the selection bias of online surveys and added evidence for the impact based on the amount of FBIH citizens online:

Finally, it remains to mention the limitations of these pandemic monitoring surveys in the FBIH. Online samples hold a potential risk of bias because they reach only the people who have access to the internet. Even if there is a high level of internet usage (73.2% in 2020, 75.7% in 2021, and 78.7% in 2022 (53)), this selection bias might be higher in older generations, so all effects where age is relevant would need to be replicated. Additional telephone surveys or household interviews might increase further insights about the effect size of this sampling bias and include underrepresented groups in the data set. Even if this additional sampling strategy was no option for this monitoring survey, future monitorings could include mixed methods in sampling. On the other hand, advantages of online surveys included that the samples were drawn to be representative and were collected in a short time period, reaching participants all over the country. Rapid data collection was of high importance during the pandemic to prevent additional confounding due to fast-changing measures and recommendations. Further, online samples are known to produce less social desirability biases (54), and especially when strict measures and recommendations were in place, social desirability might have biased the responses in interview formats. Despite these limitations, the data collected were robust enough to contribute directly to the COVID-19 response in the FBIH in communications and messaging, interventions, programs, and policy. (p.26-27, p. 460-483)

**- The discussion of policy acceptance and trust (lines 397-411) is insightful but could benefit from more elaboration on the “complex mediating role” of trust. How does this complexity play out in your findings? Could more specific examples be provided from the survey data?**

>> We did now integrate a more detailed finding of the sources we referred to, and describe in more detail the covariation of trust and policy that we can see in our data:

For instance, Guglielmi et al. (46) show how institutional confidence and specific political support influence public acceptability of containment measures in Italy, suggesting that trust in institutions can either facilitate or hinder compliance depending on political contexts. In the FBIH pandemic monitoring here, we can see that the values for trust and policy fairness decreased to the same amount from wave 1 to wave 3, but we do not see that their increase is parallel in the last 2 waves. This evidence indeed points to trust as playing an important (albeit complex) mediating role in policy acceptance (46, 47). (p. 25-26, l. 443-448)

Reviewer #2:

**The author used very simple English, and the narrative of the paper is explicit with facts.**

**The methodology was appropriate for the study.**

**The statistical analyses helped in controlling for some cofounders and biases that could have arisen from dropout of**

**some study participants.**

**The author stated limitations to the study and this guides in interpretation of the results with caution especially in relation to age and access to internet.**

>> We thank Reviewer #2 for this very positive evaluation of our manuscript.
